# Liposomal doxorubicin improves radiotherapy response in hypoxic prostate cancer xenografts

**DOI:** 10.1186/1748-717X-6-135

**Published:** 2011-10-07

**Authors:** Eirik Hagtvet, Kathrine Røe, Dag R Olsen

**Affiliations:** 1 Department of Radiation Biology, Institute for Cancer Research, The Norwegian Radium Hospital, Oslo University Hospital, P. O. Box 4953 Nydalen, 0424 Oslo, Norway; 2 Institute of Clinical Medicine, University of Oslo, Oslo, Norway; 3 Faculty of Mathematics and Natural Sciences, University of Bergen, Bergen, Norway

## Abstract

**Background:**

Tumor vasculature frequently fails to supply sufficient levels of oxygen to tumor tissue resulting in radioresistant hypoxic tumors. To improve therapeutic outcome radiotherapy (RT) may be combined with cytotoxic agents.

**Methods:**

In this study we have investigated the combination of RT with the cytotoxic agent doxorubicin (DXR) encapsulated in pegylated liposomes (PL-DXR). The PL-DXR formulation Caelyx^® ^was administered to male mice bearing human, androgen-sensitive CWR22 prostate carcinoma xenografts in a dose of 3.5 mg DXR/kg, in combination with RT (2 Gy/day × 5 days) performed under normoxic and hypoxic conditions. Hypoxic RT was achieved by experimentally inducing tumor hypoxia by clamping the tumor-bearing leg five minutes prior to and during RT. Treatment response evaluation consisted of tumor volume measurements and dynamic contrast-enhanced magnetic resonance imaging (DCE MRI) with subsequent pharmacokinetic analysis using the Brix model. Imaging was performed pre-treatment (baseline) and 8 days later. Further, hypoxic fractions were determined by pimonidazole immunohistochemistry of excised tumor tissue.

**Results:**

As expected, the therapeutic effect of RT was significantly less effective under hypoxic than normoxic conditions. However, concomitant administration of PL-DXR significantly improved the therapeutic outcome following RT in hypoxic tumors. Further, the pharmacokinetic DCE MRI parameters and hypoxic fractions suggest PL-DXR to induce growth-inhibitory effects without interfering with tumor vascular functions.

**Conclusions:**

We found that DXR encapsulated in liposomes improved the therapeutic effect of RT under hypoxic conditions without affecting vascular functions. Thus, we propose that for cytotoxic agents affecting tumor vascular functions liposomes may be a promising drug delivery technology for use in chemoradiotherapy.

## Background

During tumor growth abnormal tumor vasculature frequently fails to supply sufficient levels of oxygen to tumor tissue, resulting in various degrees of hypoxia [1,2]. Tumor hypoxia is known to cause treatment resistance and to promote metastatic disease progression [3-5]. To improve radiotherapy (RT) efficacy of radioresistant tumors, several approaches have been suggested [6,7]. One strategy is to combine conventional cytotoxic agents with RT to increase the therapeutic effects, i.e. chemoradiotherapy (CRT) [8,9].

The anthracycline chemotherapeutic drug doxorubicin (DXR) has been demonstrated to enhance the therapeutic effect of RT [10-13], presumably by preventing cells from repairing radiation-induced DNA damage [11-13]. DXR has also reportedly enhanced the effect of RT under experimental *in vitro *hypoxic conditions [14].

By encapsulating DXR in liposomes, DXR accumulation in the heart is reduced, resulting in less cardiac toxicities compared to conventional DXR [15,16]. Abnormal tumor vasculature also favors accumulation of liposomes due to the enhanced permeability retention effect [17]. Moreover, by incorporating polyethylene glycol (PEG) in the liposomal membrane, clearance by the cells of the reticulo-endothelial system is reduced, resulting in prolonged circulation time [18].

Liposomes accumulated in the tumor may act as depots for sustainable drug release, making them particularly beneficial during a course of CRT, since daily drug dosing would be needless [19]. Moreover, as liposomes avoid accumulation in healthy tissue, radiation enhancement may primarily be located to tumors, reducing toxicities in neighboring healthy tissues [19,20]. Pegylated liposomal DXR (PL-DXR) has been shown to increase the effect of RT in preclinical studies [19,21]. Promising results have been demonstrated in clinical studies in sarcoma [20], as well as in locally advanced non-small cell lung cancer and head and neck cancer [22]. In prostate cancer, anthracyclines as free doxorubicin and epirubicin alone have shown to have a palliative effect on patients with incurable, metastatic, hormone-refractory prostate cancer [23]. However, according to our knowledge no clinical investigations have reported on the combined use of anthracyclines and RT. Recognizing the impact of tumor hypoxia in prostate cancer disease progression and treatment resistance [3], the combination of anthracyclines with RT to increase radiosensitivity of hypoxic tumor regions may represent a potential therapeutic strategy for advanced prostate cancer.

The objective of this study was to evaluate the potential therapeutic benefit of administering PL-DXR (Caelyx^®^) to tumor-bearing mice receiving RT under hypoxic, radioresistant conditions. Therapy-mediated changes in tumor vascular functions and tumor hypoxia were assessed by dynamic contrast-enhanced magnetic resonance imaging (DCE MRI) and pimonidazole immunohistochemistry, respectively.

## Methods

### Materials

The PL-DXR product Caelyx^® ^was supplied by the pharmacy at the Norwegian Radium Hospital, Oslo, Norway (European distributor; Schering-Plough). Pimonidazole hydrochloride was supplied by Natural Pharmacia International, Inc., Burlington, MA, USA, and the contrast agent Dotarem^® ^was from Laboratoire Guerbet, Paris, France. Dako EnVision™+ System-HRP (DAB) was supplied by Dako Corporation, DA, USA.

For anaesthesia of mice a mixture of 2.4 mg/ml tiletamine and 2.4 mg/ml zolazepam (Zoletil^® ^vet, Virbac Laboratories, Carros, France), 3.8 mg/ml xylazine (Narcoxyl^® ^vet, Roche, Basel, Switzerland) and 0.1 mg/ml butorphanol (Torbugesic^®^, Fort Dodge Laboratories, Fort Dodge, IA, USA) was prepared and used.

### Experimental animals

Male athymic nude Balb/c mice were provided by the Department of Comparative Medicine (animal facility), Oslo University Hospital. The androgen-sensitive CWR22 xenograft, originating from a human, primary prostate carcinoma [24], was serially transplanted between mice. In brief, by blunt dissection through a skin incision tumor fragments (~2 × 2 × 2) mm^3 ^were subcutaneously implanted on the upper leg (proximal to the knee joint) of 4-5 weeks old mice. The skin incision was sealed with topical skin adhesive. Approximately three weeks later a tumor xenograft of 5 - 10 mm in diameter developed. The mice were housed in transparent boxes with bedding material, fed *ad libitum *and kept under specific pathogen-free conditions. The temperature and relative humidity were kept constant at 20 - 21°C and 60%, respectively. At the end of the experiments all mice were euthanized by cervical dislocation. All procedures were performed according to protocols approved by the National Animal Research Authority and carried out in compliance with the European Convention for the Protection of Vertebrates Used for Scientific Purposes.

### Radiotherapy

RT was delivered at a dose of 2 Gy/day for five consecutive days (at experiment days 1 - 5) using a ^60^Co source (Mobaltron 80, TEM instruments, Crawley, UK) with a dose rate of 0.8 Gy/min. The mice were located in a custom designed vicryl tube with an opening for the tumor bearing leg to be stretched out and fixated horizontally. During the procedure only the tumor bearing leg was extended into the radiation field, limiting radiation exposure to the remaining body. The procedure was performed under sedation induced by 0.05 ml of anesthetic agent.

### Hypoxic radiotherapy

Tumor hypoxia was experimentally induced by placing the mice in a vicryl tube. A rubber band was clamped around the leg of the mouse, proximal to the xenograft. The rubber band was left on for five minutes prior to and during RT (at experiment days 1 - 5). During clamping the leg of the mouse temporary turned bluish, indicating stagnation of blood circulation with concurrent induction of acute hypoxia. The discoloration disappeared rapidly following removal of the rubber band and no mice became lame or experienced any adverse effects from the clamping. The procedure was performed under sedation induced by 0.05 ml of anesthetic agent.

### PL-DXR

PL-DXR was administered at a dose of 3.5 mg DXR/kg as a single i.v. bolus injection through the tail vein (at experiment day 0). The rationale for using the relatively low drug dose was to avoid reaching therapy saturation levels where any additional effect produced by hypoxic RT would not be detected.

### Monitoring of treatment response

Mice bearing tumor xenografts sized 5 - 10 mm in diameter were randomly allocated into different experimental groups of 8 - 10 tumors each (Table 1). At the start of the experiment all mice were imaged by DCE MRI with subsequent i.v. administration of PL-DXR to mice designated to the PL-DXR groups. RT treatment began 24 hrs later, enabling sufficient time for liposomal tumor accumulation. During daily RT sessions all mice, regardless of experimental group, were sedated. To assess therapy-induced changes in tumor vascular function all mice were subjected to an identical imaging protocol 8 days after the pre-treatment DCE MRI.

**Table 1:** Overview of treatments administered to the different experimental groups

Experimental groups	Treatment
Control	No treatment

PL-DXR	3.5 mg DXR/kg (day 0)

PL-DXR + hypoxic RT	3.5 mg DXR/kg (day 0) + clamping + 2 Gy/day for 5 days (day 1 - day 5)

RT	2 Gy/day for 5 days (day 1 - day 5)

Hypoxic RT	Clamping + 2 Gy/day for 5 days (day 1 - day 5)

Tumor volumes were estimated after measuring the tumors' shortest and longest diameters with four days intervals using a digital caliper (Model B220S, Kroeplin, Schlüchtern, Germany). The tumor volume was calculated according to the formula (π/6)*length^2^*width [25]. The tumor growth delay (TGD) in days for tumors to reach a 3-fold increase in relative tumor volume, *i.e*. treated tumors compared to control tumors; *TGD*_*V3*_, was found for all experimental groups.

### DCE MRI acquisitions

MRI acquisitions were performed as previously described [26], using a 1.5 T GE Signa LS scanner (GE Medical Systems, Milwaukee, WI), and a dedicated MRI mouse coil [27]. Prior to MRI, a heparinized 24G catheter attached to a cannula containing 0.01 ml/g body weight contrast agent (Dotarem^®^, diluted in heparinized saline to 0.06 M) was inserted into the tail vein of the mice. The mice were placed in an adapted cradle and put into the coil, before being placed in the scanner. During image acquisition, the temperature of the mouse was maintained at 38°C. First, the tumor was localized using axial fast spin-echo (FSE) T2-weighted (T2W) images (echo time (TE_eff_) = 85 ms, repetition time (TR) = 4000 ms, echo train length (ETL) = 16, image matrix (IM) = 256 × 256, field-of-view (FOV) = 4 cm, slice thickness (ST) = 2 mm). Second, DCE MRI was obtained with a dynamic fast spoiled gradient-recalled (FSPGR) T1W sequence (TE = 3.5 ms, TR = 180 ms, IM = 256 × 128, FOV = 6 cm, ST = 2 mm, and flip angle (FA) = 80°). Following 5 baseline T1W image acquisitions, contrast kinetics were investigated by injecting the contrast agent during 3 seconds and performing 20 minutes of post-contrast imaging. The time resolution was 12 seconds and the reconstructed voxel size was 0.23 × 0.23 × 2 mm^3^.

### DCE MRI analysis

Image analysis was performed using in-house developed software in IDL (Interactive Data Language v 6.2, Research Systems Inc., Boulder, CO). For the central slice of each tumor, a region of interest (ROI) was manually traced in T1W images, excluding surrounding skin and connective tissue. The time-dependent relative signal intensity, *RSI(t)*, was calculated for each image voxel according to Equation 1.

(1) $$RSI\left(t\right)=\frac{SI\left(t\right)-SI\left(0\right)}{SI\left(0\right)}$$

where *SI(0) *refers to the pre-contrast signal intensity and *SI(t) *the post-contrast signal intensity in the voxel at time *t*. To subsequently enable comparison of all tumors in the experiment, it was ensured that all post-contrast images were initiated after a 3 seconds injection of contrast agent. By using the MRI scanner's recorded image information any deviations from these 3 seconds could be corrected by adjusting the time-axis of the post-contrast image set. Pharmacokinetic modeling was performed using the Brix model [28], with the *RSI(t) *for each voxel as input. The Brix model is a two-compartment pharmacokinetic model where the contrast agent is assumed to distribute between two individually well-mixed compartments; the blood plasma and the extracellular extravascular space (EES) in the tumor. The i.v. injected contrast agent is transported into the tumor by perfusion, where it diffuses between the plasma and the EES, before being eliminated at a constant rate.

Using the *RSI(t) *for each voxel in the tumor ROI, the Brix model (equation 2) was fitted using the Levenberg-Marquardt least-squares minimization method (MPFIT; http://purl.com/net/mpfit) [29].

(2) $$RSI\left(t\right)=\frac{Ak_{ep}}{k_{el}k_{ep}}\left(e^{-k_{ep}t}-e^{-k_{el}t}\right)$$

where the parameter *k*_*ep *_is the rate constant between plasma and EES, *k*_*el *_the clearance rate of contrast agent from plasma, and *A *an amplitude parameter related to the size of the EES.

### Immunohistochemistry of tumor hypoxia

In addition to the mice subjected to DCE MRI, parallel groups of mice were followed to harvest tumor tissue at the same time-point as the day 8 MRI acquisitions. Mice designated to immunohistochemistry examination received identical treatments as mice used for tumor growth assessment and DCE MRI (Table 1), with each group containing 8 tumors. Hypoxia was determined by injecting 80 mg/kg pimonidazole hydrochloride (1-[(2-hydroxy-3-piperidinyl)propyl]-2-nitroimidazole hydrochloride, dissolved in saline i.p. One hour later euthanasia was performed by cervical dislocation and tumors were excised and preserved in phosphate-buffered 4% formalin until tissue sectioning. Tumor hypoxia was detected using a peroxidase-based immunostaining method. In brief, tissue sections were stained using the Dako EnVision™+ System-HRP (DAB) (K4011) and Dakoautostainer. Deparaffinization and unmasking of epitopes were performed using PT-Link (DAKO) and EnVision™ Flex target retrieval solution, with high pH. To block endogenous peroxidase, sections were treated with 0.03% hydrogen peroxide for 5 minutes. The preparations were incubated 30 minutes with polyclonal rabbit antibodies to pimonidazole-protein adducts (1:10000 dilution). The sections were then incubated with peroxidase-labeled polymer conjugated to goat anti-rabbit secondary antibodies for 30 minutes. The tissue sections were stained for 10 minutes with 3'3-diaminobenzidine tetrachloride (DAB) and counterstained with haematoxylin, dehydrated and mounted in Diatex.

### Statistical analysis

By means of a multiple regression procedure differences in tumor growth between the experimental groups were operationally represented by three between group comparisons; 1) comparing the RT group with the hypoxic RT group, 2) comparing the RT group with the PL-DXR group and finally, 3) comparing the hypoxic RT group with the PL-DXR + hypoxic RT group. Tumor growth was represented by linear and quadratic developmental trends.

Group differences in DCE MRI parameters and hypoxic fractions were analyzed by student's *t*-tests, and the Pearson correlation (*r*) test analyzed whether correlations between variables were significant using SPSS 16.0 (SPSS, Cary, NC). A significance level of 5% was used for all statistical analyses.

## Results

### Tumor growth

**Figure 1:**
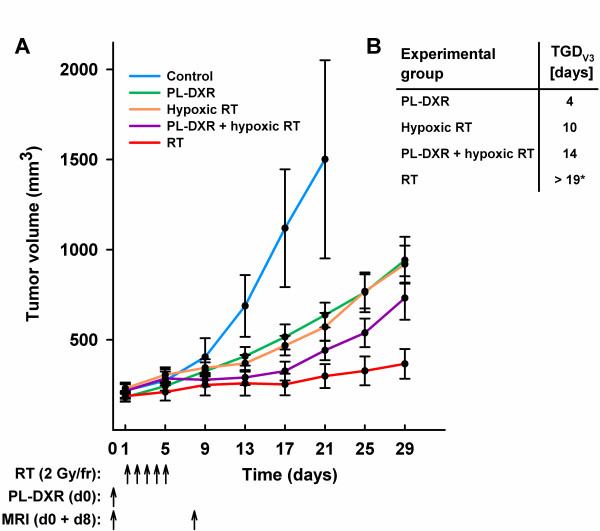
**Tumor growth patterns for the experimental groups**. Presented as mean ± SEM (n = 8 - 10 per group). The control group was removed from the study at day 21 due to tumor diameters exceeding 20 mm (A). Tumor growth delays as mean number of days for tumors to reach a 3-fold increase in relative tumor volume for each of the treatment groups versus the control group, *TGD*_*V3*_. *The RT group did not reach 3-fold increase in relative volume at day 29; thus the *TGD*_*V3 *_is more than 19 days (B).

Tumor volume measurements were performed with four days intervals for 29 days, except for the control group where mice were euthanized at day 21 when the tumor diameters exceeded 20 mm, i.e. in accordance with internal regulations for animal experiments. Based on the 21 days observation period, the tumor growth of the control group was significantly faster compared to all treatment groups (p < 0.050). The differences in tumor growth between the remaining groups were analyzed on the basis of the 29 days observation period. Based on quadratic developmental trends the hypoxic RT group showed significantly less therapeutic effect than the normoxic RT group (comparison 1, p = 0.006). The group receiving PL-DXR also presented significantly less therapeutic effect than the RT group (comparison 2, p = 0.008). Interestingly, tumor growth in the PL-DXR + hypoxic RT group was significantly reduced compared to the hypoxic RT group (comparison 3, p = 0.004). Tumor growth patterns are portrayed in Figure 1. No adverse effects, including skin toxicities, were observed in any of the experimental groups.

### Treatment monitoring using DCE MRI

Following Brix modeling of contrast kinetics, parametric images of *A, k*_*el *_and *k*_*ep *_were produced. The *k*_*ep *_parameter is a parameter being estimated based on the initial increase in the *RSI *curve, which is reflecting the in-wash of contrast agent from plasma to EES. Due to these tumors' high permeability and/or high perfusion, this initial increase was very steep, consequently precluding the Brix model to reliably estimate mean tumor values of *k*_*ep *_for subsequent intra- and intergroup comparisons. The *k*_*ep *_parameters were therefore excluded. Also, due to unsuccessful injection of contrast agent or technically related issues, some of the tumors in the experiment were excluded from subsequent pharmacokinetic analysis. Further, some of the tumors were too small to enable reliable DCE MRI analysis. The exact number of tumors that underwent MRI and image analysis is indicated in all relevant figures onwards.

**Figure 2:**
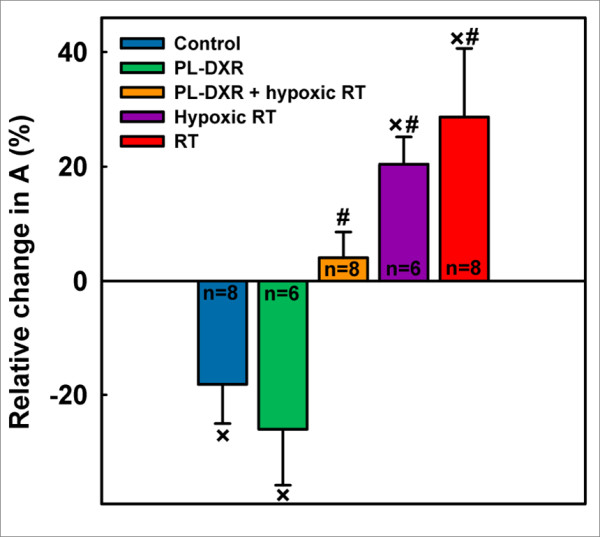
**Relative change in the *A *parameter (mean ± SEM) from day 0 to day 8 for the experimental groups**. 3.5 mg/kg PL-DXR was administered after pre-treatment DCE MRI. RT was delivered at a dose of 2 Gy/day for 5 consecutive days, starting 24 hours after the pre-treatment DCE MRI. Hypoxia was induced by clamping the tumor-bearing leg 5 minutes prior to and during RT. Significant differences (p < 0.050) to the control or PL-DXR + hypoxic RT groups are indicated with # or ×, respectively.

In Figure 2, the mean group relative change in the *A *parameter; an amplitude parameter related to the size of the EES [28], from day 0 to day 8 is presented. A reduction in the *A *parameter was observed for both the control (18%) and the PL-DXR (26%, p = 0.030) groups. All groups receiving radiation experienced a relative increase from day 0 to day 8, being 4% in the PL-DXR + hypoxic RT group (not significant), 20% (p = 0.002) in the hypoxic RT group and 29% (p = 0.046) in the RT group. No significant intergroup difference in the *A *parameter was observed when comparing the control tumors with tumors treated with PL-DXR. However, all groups receiving radiation experienced a significant increase in the *A *parameter compared to the control group; PL-DXR + hypoxic RT (p = 0.019), hypoxic RT (p = 0.001) and RT (p = 0.006). Additionally, the group receiving PL-DXR + hypoxic RT also experienced an increase in the *A *parameter compared to PL-DXR (p = 0.026) and a decrease compared to hypoxic RT (p = 0.025) and RT (p = 0.049).

**Figure 3:**
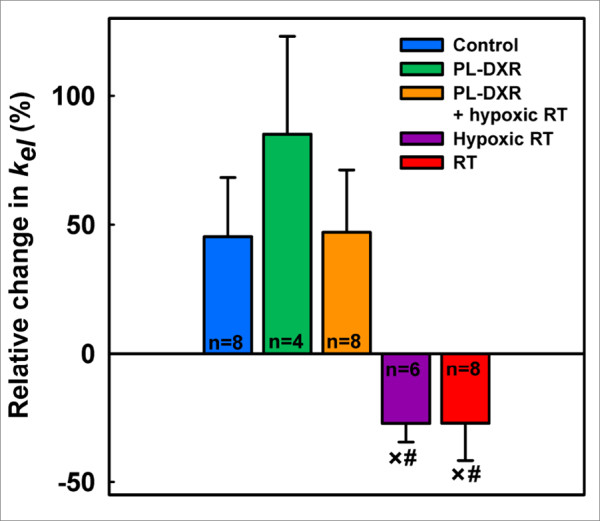
**Relative change in the *k***_***el ***_**parameter (mean ± SEM) from day 0 to day 8 for the experimental groups**. 3.5 mg/kg PL-DXR was administered after pre-treatment DCE MRI. RT was delivered at a dose of 2 Gy/day for 5 consecutive days, starting 24 hours after pre-treatment DCE MRI. Hypoxia was induced by clamping the tumor-bearing leg 5 minutes prior to and during RT. Significant differences (p < 0.050) to the control or PL-DXR + hypoxic RT groups are indicated with # or ×, respectively.

In Figure 3, the mean group relative change in the *k*_*el *_parameter, reflecting the clearance rate of contrast agent from plasma [28], from day 0 to day 8 is presented. Three groups experienced an increase in *k*_*el*_, being 45% in the control group, 85% in the PL-DXR group and 47% in the PL-DXR + hypoxic RT group. Due to large intragroup variations, these increases were not significant. Both the hypoxic and normoxic RT groups experienced a 27% decrease in the *k*_*el *_parameter with the change in the hypoxic RT group being significant (p = 0.007). No intergroup differences in the *k*_*el *_parameter were observed when comparing the control tumors with the tumors that received PL-DXR or PL-DXR + hypoxic RT. However, both the hypoxic RT group and the RT group experienced significant reductions in the *k*_*el *_parameter compared to the control group, (p = 0.015 and p = 0.020, respectively).

### Immunohistochemistry of tumor hypoxia

**Figure 4:**
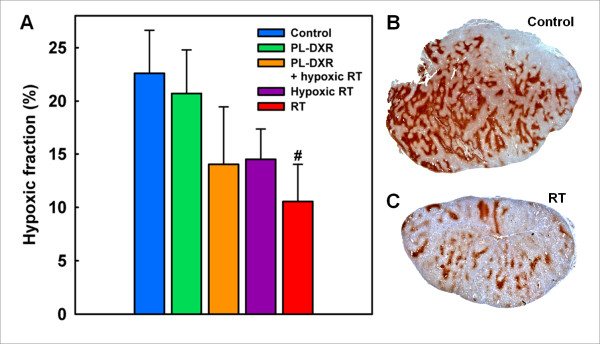
**Hypoxic fractions in the experimental groups at day 8 assessed from pimonidazole immunohistochemistry of tumor tissue sections**. Group mean and SEM are shown, with n = 8 per group. 3.5 mg/kg PL-DXR was administered 24 hours prior to RT. RT was delivered at a dose of 2 Gy/day for 5 consecutive days. Hypoxia was induced by clamping the tumor-bearing leg 5 minutes prior to and during RT. A significant difference (p < 0.05) to the control group is indicated with # (A). Representative images of pimonidazole-stained sections of an untreated tumor (B), and an irradiated tumor (C), respectively.

Parallel to tumor growth and DCE MRI studies identically treated groups of tumors were excised and used to assess tumor hypoxia by performing pimonidazole immunohistochemistry of tumor tissue excised at day 8, coinciding with the time-point of post-treatment MRI acquisitions. Figure 4A presents the hypoxic fractions of the different experimental groups. The mean hypoxic fractions were 23% for the control tumors, 21% for tumors treated with PL-DXR alone, 14% for the tumors receiving both PL-DXR and hypoxic RT, 15% for tumors receiving hypoxic RT, and 11% for tumors receiving RT. Compared to the control group, only the RT group presented significantly reduced hypoxic fractions (p = 0.041). Figure 4B and 4C show examples of pimonidazole staining in representative untreated and irradiated tumors, respectively.

## Correlations

**Figure 5:**
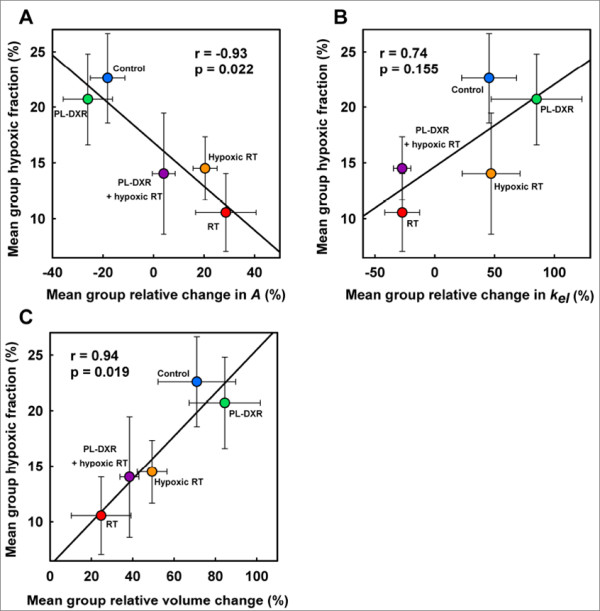
**Hypoxic fractions versus DCE MRI parameters and tumor volumes**. Correlations between the mean group hypoxic fractions (%) at day 8 versus the mean group relative change in the *A *parameter from day 0 to day 8 (%) (A), the mean group relative change in the *k*_*el *_parameter from day 0 to day 8 (%) (B), and the relative change in mean group tumor volumes from day 0 to day 9 (%) (C), respectively.

Figure 5 shows the correlations between the mean group hypoxic fractions at day 8 (%) versus the mean group relative change in the *A *parameter from day 0 to day 8 (%) (Figure 5A), the mean group relative change in the *k*_*el *_parameter from day 0 to day 8 (%) (Figure 5B), and the mean group relative change in tumor volumes from day 0 to day 9 (%) (Figure 5C), respectively. The mean group hypoxic fractions showed a strong negative correlation to the mean group relative change in the *A *parameter from day 0 to day 8 (*r* = -0.93, p = 0.022), a weaker and insignificant positive correlation to the mean group relative change in the *k*_*el *_parameter from day 0 to day 8 (*r *= 0.74, p = 0.155), and a positive correlation to the mean group tumor volume change from day 0 to day 9 (r = 0.94, p = 0.019).

**Figure 6:**
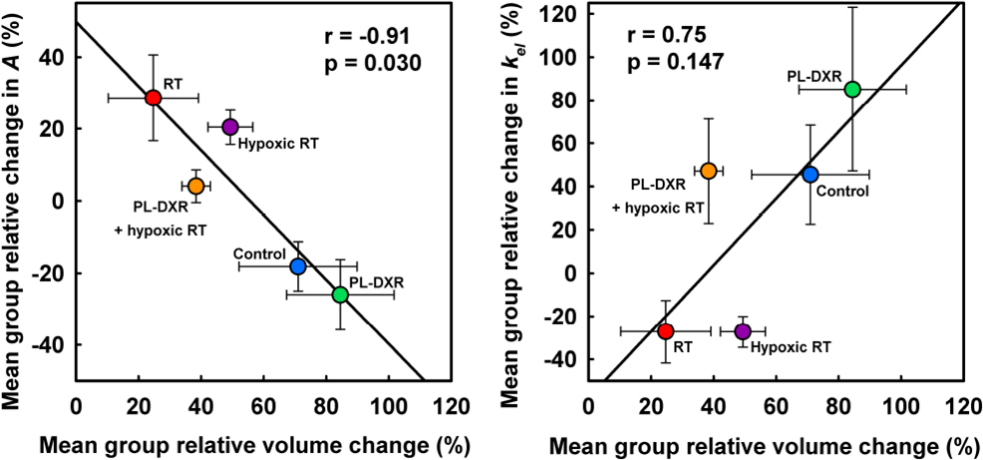
**Tumor volume changes versus DCE MRI parameter changes**. Correlations between the mean group relative change in tumor volumes (%) from day 0 to day 9 (%) versus the mean group relative change in the *A *parameter from day 0 to day 8 (%) (A), and the mean group relative change in the *k*_*el *_parameter from day 0 to day 8 (%) (B), respectively.

Figure 6 shows the correlations between the mean group relative change in tumor volumes from day 0 to day 9 (%) versus the mean group relative change in the *A *parameter from day 0 to day 8 (%) (Figure 6A) and the mean group relative change in the *k*_*el *_parameter from day 0 to day 8 (%) (Figure 6B), respectively. Mean group tumor volume change correlated negatively to the mean group relative change in the *A *parameter (r = -0.91, p = 0.030) from day 0 to day 8, and positively, but not significantly, to the mean group relative change in the *k*_*el *_parameter (r = 0.75).

## Discussion

Tumor hypoxia prevent effective RT [3-5], and several strategies to improve RT efficacy under hypoxic conditions have been described [6,7]. The ability of PL-DXR to enhance the therapeutic effect of fractionated and single dose RT has previously been reported [19,21]. In the current study we demonstrated that PL-DXR improves the therapeutic effect of RT also under hypoxic conditions. Moreover, as it is important to develop strategies to monitor treatment responses non-invasively, DCE MRI appears to be promising for this purpose.

The current PL-DXR formulation accumulates slowly in tumors, reaching peak levels 2-3 days post injection in tumor xenograft models [30,31]. Consequently, substantial levels of PL-DXR in the tumors during the five days of RT were expected. Any RT-mediated changes in tumor vascular functions that could interfere with tumor liposome accumulation was expected to be minimal as RT previously has reported to not alter liposomal tumor uptake [32,33].

Free DXR is reported to decrease tumor blood flow [34,35], subsequently reducing the oxygen levels in tumors. In contrary, PL-DXR has been suggested to normalize tumor vasculature [36]. In the current study there was no significant difference between the control and the PL-DXR group in any of the DCE MRI derived kinetic parameters or hypoxic fractions, suggesting that PL-DXR did not alter vascular functions. Still, tumor growth was significantly inhibited indicating that PL-DXR may exert tumoricidal effects without interfering with tumor blood circulation. This feature is highly beneficial with respect to subsequent RT since well oxygenated and vascularized tumors more likely respond better to RT.

In contrary, RT induced changes in the tumor vasculature both in the hypoxic and normoxic tumors, as measured by an increase in the *A *parameter. This alternation may be related to an increased interstitial volume, and a reduced elimination rate of contrast agent, as indicated by the *k*_*el *_parameter. The increase seen in the *A *parameter may be related to radiation-induced necrosis and/or edema, and thus increased interstitial volume. Further, an increase in the *A *parameter may reflect disrupted membranes increasing the extracellular volume due to elevated membrane permeability. Finally, the observed reductions in the *k*_*el *_parameter may reflect radiation-induced endothelial cell death, making clearance of contrast agent less effective. Interestingly, when hypoxic RT was administered in combination with PL-DXR these changes became less evident, indicating that PL-DXR reduced some of the vascular effects caused by RT in hypoxic tumors.

Based on the pharmacokinetic theory behind the Brix model the amplitude parameter *A *is related to the size of the EES [28]. Here we show that the changes in the *A *parameter from day 0 to day 8 were significantly correlated to tumor hypoxic fractions (Figure 5A). A similar relation has also been found in a clinical DCE MRI study of cervical cancer, where a positive correlation between the *A *parameter and oxygen levels, as measured by Eppendorf pO_2 _histography, was evidenced [37]. This may suggest the *A *parameter as a candidate biomarker of tumor hypoxia, for further investigation. The *k*_*el *_parameter is theoretically reflecting the clearance rate of contrast agent from plasma [28], which is affected by the functionality of the tumor vasculature. Compared to the *A *parameter, our results showed that the *k*_*el *_parameter correlated less to hypoxia (Figure 5B). However, hypoxic fractions correlated significantly (Figure 5C) with tumor volume changes and may explain why the measured hypoxic fractions were highest in the control tumors and lowest in the tumors receiving the most effective treatments. Hypoxia and tumor size have also previously been demonstrated to correlate strongly [38].

During the last years, several imaging modalities have been investigated for their possible ability to provide non-invasive biomarkers of tumor hypoxia. If such biomarkers can be identified and validated, they are likely to provide important consequences in personalized cancer treatment, for example in detection of treatment-resistant tumors requiring alternative therapeutic strategies, for delivering intensified radiotherapy to hypoxic tumor regions, and as a means to monitor the response to therapies. In this respect, particularly positron emission tomography (PET) using various radiotracers aiming to detect tumor hypoxia [39], and DCE MRI [40], are clinically feasible and promising tools which have been employed in preclinical and clinical studies. However, the potential of providing more quantitative measures by applying pharmacokinetic models in data analysis is currently less investigated, warranting further studies. The benefits of using MRI compared to PET are particularly the avoidance of ionizing radiation exposure and injection of radioactive sources, as well as being a more cost-effective imaging modality.

The treatment-induced changes in the *A *parameter correlated significantly and negatively to tumor volume changes (Figure 6A), and changes in the *k*_*el *_parameter correlated strongly and positively, although not significantly, to these volume changes (Figure 6B). This is promising with respect to developing DCE MRI and pharmacokinetic image analysis as tools for non-invasive monitoring of therapeutic effects.

The presence of oxygen in tumors exposed to RT is crucial because oxygen 1) enhance the yield of radiation-induced radicals and thus DNA damage, and 2) prevent repair of induced DNA damage by fixation of the damage [41]. DXR enhances the therapeutic effect of RT presumably by preventing cells from repairing radiation-induced DNA damage [11-13]. DXR may therefore resemble the effect of oxygen in tumors exposed to RT. Hypoxia is a common feature amongst most solid, clinical tumors [42]. Overcoming hypoxia by administration of radiosensitizing drugs may nevertheless be of limited success as their supply to hypoxic regions commonly are hampered by inadequate vascularization. Liposomal DXR seems however to have a positive effect on the tumor vascular functions as shown in this study.

## Conclusion

The present study shows that PL-DXR improves the therapeutic effect of RT under hypoxic conditions and that PL-DXR does not affect tumor vascular functions. Interestingly, PL-DXR appeared to reduce some of the vascular alterations induced in hypoxic tumors by RT. Hence, for drugs that affect tumor vascular functions liposomes may be a promising drug delivery technology for use in CRT.

## Competing interests

The authors declare that they have no competing interests.

## Authors' contributions

EH participated in study design, carried out the animal experiments, MRI data acquisition, immunohistochemistry analysis and wrote the manuscript. KR participated in study design, designed MRI protocols, analyzed MRI data, discussed the data, and participated in writing the manuscript. DRO participated in study design, data discussion and revision of the manuscript. All authors read and approved the final manuscript.
